# Reviews and Bibliographical Notices

**Published:** 1882-05

**Authors:** 


### REVIEWS AND BIBLIOGRAPHICAL NOTICES.

Pocket Book of Physical Diagnosis. By Dr. Ed. T. Bruen. Presley Blakiston, Phila., 1881. $2. This excellent manual of physical diagnosis is devoted to a consideration of the investigation of diseases of the lungs, pleuræ, heart and pericardium, with chapters on malignant diseases of the mediastinum, aneurismal diseases of the arterial system, and the sphygmograph.

It may seem hypercritical, but in the interest of students (for whom the book is written) we should call attention to a few defects. The effort at condensation has in some places been carried to excess, so that the author's meaning becomes obscure. A purist might say that the "diagnosis of early phthisis" should be the "early diagnosis of phthisis," and on page 35 it would seem more in accordance with the author's plan to insert the sub-title *vocal resonance*. The print and make-up of the book are good, and the few diagrams given are useful.

The sphygmograph he concludes is really useful to the physiologist, but is only valuable clinically when combined with the other methods of investigation.

The author says that a presystolic murmur may occur either from obstruction or roughening; to distinguish between the two he notes that if true stenosis exists there will be enlargement (hypertrophy with dilatation) of the left auricle and the murmur is heard over the auricular area. Dr. Flint, in an article published since this work was written (*Am. J. Med. Sci.* Apr. '82, p. 442), divides the presystolic murmur into two: a rough and a soft murmur; he shows that a mitral obstructive lesion may exist without a murmur being present, and that we cannot absolutely tell from the character of the murmur whether an obstruction or simple roughening is present.

The book is a very useful one, and we can heartily recommend it to both practitioners and students.

*The Health Service of a State.* By George E. Ranney, M.D. Pp. 14.
*Consumption: Is it a Contagious Disease? What can be done to prevent its ravages?* By Bela Cogshall, M.D. Pp. 12.

The above are two articles reprinted from the Annual Report of the State Board of Health of Michigan for 1881. Dr. Ranney's paper gives a summary of the most important work done by the Michigan State

Board. From an examination of this it is plain that the Michigan board is the most practical organization of its character in the United States. The titles of 135 sanitary tracts and short articles are given, which have been published by the board and distributed to the people throughout the State. There are articles of 19 pages on Baths and Bathing, Cerebro-spinal Meningitis 78 pages, various contributions to the study of the spread of diphtheria 53 pages, Drainage 25 pages, Resuscitation of the Drowned 22 pages, Periodic Fevers 21 pages, Heredity 18 pages, Disposal of Excreta 22 pages, the Influence of Occupations upon Health 16 pages, Ozone 37 pages, Vaccination 19 pages, and numerous shorter ones on equally practical subjects. The paper of Dr. Cogshall is one of these tracts and in a brief compass summarizes the latest views, up to the date of its reading, of the etiology of tuberculosis. The Wolverine State is far ahead of her sisters in practical sanitary work.

*The Popular Science Monthly* for May, beginning a new volume, is filled as usual with interesting scientific contributions. The papers of most interest to physicians are one, on "Color-blindness and Color-perception" by Dr. Swan M. Burnett, and one giving some account of the culture of the Cinchona tree in India, by Dr. O. R. Batcheler. Herbert Spencer, Alfred Russell Wallace, Francis Galton, and Dr. F. L. Oswald also contribute articles of value and interest. A portrait and sketch of Sir John Lubbock, the eminent naturalist, anthropologist, statesman and banker embellishes the current number of the magazine.

*The Canada Health Journal.* Vol. 5, No. 8. April 1882 Published at Toronto, Can. After a suspension of several months we are glad to see this valuable journal among our exchanges again. The present number contains one of Dr. B. W. Richardson's inimitable lectures, its title being "The Seed-time of Health." We wish our contemporary abundant prosperity.

